# Three-Dimensional Volumetric Iodine Mapping of the Liver Segment Derived from Contrast-Enhanced Dual-Energy CT for the Assessment of Hepatic Cirrhosis

**DOI:** 10.3390/tomography11100109

**Published:** 2025-09-29

**Authors:** Yosuke Kawano, Masahiro Tanabe, Mayumi Higashi, Haruka Kiyoyama, Naohiko Kamamura, Jo Ishii, Haruki Furutani, Katsuyoshi Ito

**Affiliations:** Department of Radiology, Yamaguchi University Graduate School of Medicine, Ube 755-8505, Yamaguchi, Japan

**Keywords:** hepatic fibrosis, dual-energy CT, iodine mapping, extracellular volume

## Abstract

**Objective**: This study aimed to evaluate the hepatic volume, iodine concentration, and extracellular volume (ECV) of each hepatic segment in cirrhotic patients using three-dimensional (3D) volumetric iodine mapping of the liver segment derived from contrast-enhanced dual-energy CT (DECT) superimposed on extracted color-coded CT liver segments in comparison with non-cirrhotic patients. **Methods**: The study population consisted of 66 patients, 34 with cirrhosis and 32 without cirrhosis. Using 3D volumetric iodine mapping of the liver segment derived from contrast-enhanced DECT superimposed on extracted color-coded CT liver segments, the volume and iodine concentration of each hepatic segment in the portal venous phase (PVP) and equilibrium phase (EP), the difference in iodine concentration between PVP and EP (ICPVP-liver—ICEP-liver), and ECV fractions were compared between cirrhotic and non-cirrhotic groups. **Results**: The iodine concentration was not significantly different in all hepatic segments between the cirrhotic and non-cirrhotic groups. Conversely, the difference in iodine concentration between PVP and EP (ICPVP-liver—ICEP-liver) was significantly smaller in the cirrhosis group than in the non-cirrhosis group for all hepatic segments (*p* < 0.001). The ECV fraction of the left medial segment was significantly higher in the cirrhosis group than in the non-cirrhotic group ([26.4 ± 7.6] vs. [23.1 ± 5.1]; *p* < 0.05). **Conclusions**: The decreased difference in iodine concentration between PVP and EP calculated from 3D volumetric iodine mapping of the liver segment using DECT may be a clinically useful indicator for evaluating patients with compensated cirrhosis, suggesting a combined effect of a reduced portal venous flow and increased interstitial space associated with fibrosis.

## 1. Introduction

Liver cirrhosis is a multifactorial condition, with a variety of etiologies including obesity, non-alcoholic fatty liver disease, heavy alcohol consumption, hepatitis B or C infection, autoimmune disease, cholestatic disease, and excessive iron and copper intake [1]. Chronic inflammation due to these etiologies results in fibrosis, as the liver parenchyma is replaced by stromal cells, such as activated astrocytes and myofibroblasts, leading to the deposition of extracellular matrix, mainly type I collagen [2]. Fibrosis is pathologically associated with microvascular short-circuiting and reduction in circulating blood volume in sinusoids. This is due to compression of portal vein branches by regenerative nodules, crushing, and dysfunction of the hepatic veins. It is also known to produce characteristic liver morphological changes in selective atrophy and compensatory enlargement in patients with cirrhosis. This phenomenon is believed to result from alterations in intrahepatic hemodynamics and fibrosis, influenced by a multitude of factors, including asymmetric anatomy of the left and right liver, central and peripheral zonal differentiation, portal perfusion, and hepatic venous drainage [3]. In a previous study, morphological changes in the liver associated with fibrosis were strongly correlated with portal vein hemodynamics, i.e., blood flow in the right branch of the portal vein tended to decrease, while that in the left branch of the portal vein increased [4].

Recent advancements have led to the development of fully automated three-dimensional (3D) volumetric computed tomography (CT) software programs [5] that generate 3D CT images of the entire liver and color-coded volumetric segmentation of each liver segment to quantitatively assess volumetric changes in the liver segment. In addition, the contrast-enhanced dual-energy CT (DECT) technique can accurately quantify the amount of iodine in the liver associated with hepatic blood flow because it illustrates the iodine distribution on an iodine map, which represents the amount of iodine per voxel [6,7]. Iodine maps are also used to measure extracellular volume (ECV) to grade liver fibrosis [6,8]. By superimposing the iodine map image with each color-coded liver segment on contrast-enhanced dynamic images, the mean iodine concentration (mgI/cm^3^) related to hepatic blood flow and ECV fraction of each liver segment can be measured on a volume basis, although such an assessment has not been reported in clinical studies.

The present study therefore evaluated the hepatic volume, iodine concentration, and ECV of each hepatic segment in cirrhotic patients using 3D volumetric iodine mapping of the liver segment derived from contrast-enhanced DECT superimposed on extracted color-coded CT liver segments in comparison with non-cirrhotic patients.

## 2. Materials and Methods

### 2.1. Study Population

**Figure 1:**
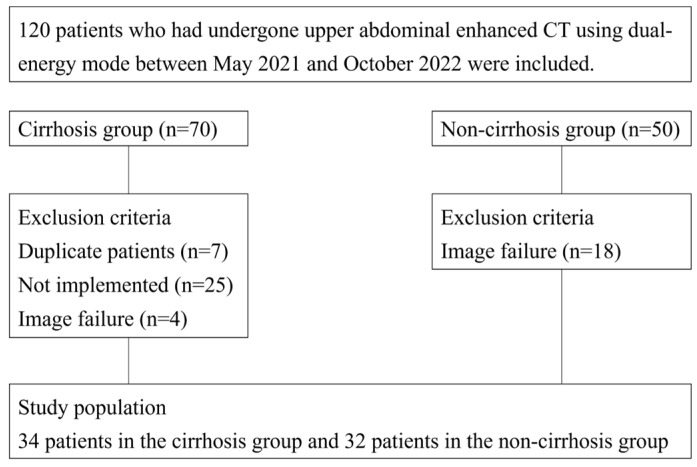
Flowchart of patient selection.

This retrospective study received institutional review board approval, and the requirement for written informed consent was waived. Our radiology database was searched to recruit patients who underwent multiphasic contrast-enhanced dynamic CT of the abdomen using dual-energy mode between May 2021 and October 2022. The following patients were excluded: those missing iodine map images (*n* = 16), those in whom the portal vein was not recognized (*n* = 1), and those for whom image data were not retrieved (*n* = 1). Thus, the final study population included 66 patients (34 men, 32 women; mean age, 65.2 ± 11.6 [range, 33–84 years]), consisting of 34 patients in the cirrhosis group and 32 patients in the non-cirrhosis group (Figure 1).

The reasons for abdominal MRI included surveillance or post-therapeutic follow-up of hepatocellular carcinoma (*n* = 30) and an evaluation of portal hypertension (*n* = 4) in the cirrhosis group as well as a further evaluation of suspected pancreatic abnormalities (e.g., pancreatic duct stenosis or cystic lesions) (*n* = 13), gastrointestinal lesions (e.g., gastric cancers) (*n* = 10), benign hepatic lesions (e.g., hemangioma) (*n* = 8), and benign biliary stricture (*n* = 1) in the non-cirrhosis group. The causes of cirrhosis included hepatitis C virus infection (*n* = 14), hepatitis B virus infection (*n* = 6), alcohol consumption (*n* = 6), nonalcoholic steatohepatitis (*n* = 5), primary biliary cholangitis (*n* = 1), and cryptogenic (*n* = 2). Patients in the cirrhosis group were identified based on a clinical diagnosis. All patients had their clinical and laboratory data consulted to determine the fibrosis-4 (FIB-4) index, albumin–bilirubin (ALBI) score, aminotransferase/platelet ratio index (APRI), and Child–Pugh classification.

### 2.2. CT Technique

All examinations were performed with a dual-source, dual-energy CT scanner (SOMATOM Force; Siemens Healthineer, Forchheim, Germany) with the following parameters: tube voltages, 100 kV and 150 kV with tin filtration; detector collimation, 128 × 0.6 mm; pitch, 0.6; gantry rotation time, 0.5 s; matrix, 512 × 512; and reconstruction thickness, 1.0 mm with no gaps using an iterative reconstruction program (adaptive model-based iterative reconstruction [ADMIRE]) at a level of 2. The tube current was controlled using automated tube current modulation (CareDose 4D).

All patients underwent unenhanced CT in the supine position. Subsequently, multiphasic contrast-enhanced dynamic CT was conducted during the arterial phase (AP), portal venous phase (PVP), and equilibrium phase (EP). Iodinated contrast material (600 mgI/kg) was injected intravenously with a fixed injection duration of 30 s (3–5 mL/s flow rate, depending on the patient’s weight), followed by a 30-mL saline flush. AP scanning was initiated automatically 20 s after a bolus-tracking system detected a trigger threshold of 100 Hounsfield units (HU) at the level of the abdominal aorta. PVP and EP scanning were started 60 and 180 s after the start of intravenous injection, respectively.

For AP, PVP, and EP CT images, using a blending factor of 0.5 (50% data of 100 kVp and 50% data of 150 kVp spectrum), blended images corresponding to 120 kVp CT were created. In addition, iodine concentration map images were generated from each phase of contrast-enhanced DECT using a post-processing software program (Syngo via version 10; Siemens Healthineers, Forchheim, Germany) to measure iodine concentrations in the AP, PVP, and EP of the liver.

### 2.3. Image Analyses

**Figure 2:**
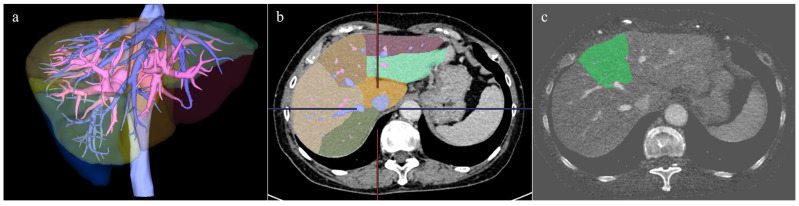
Segmentation of each liver segment based on portal vein and its branches, and an example of the fusion image with iodine map. (**a**) Extraction of large vessels such as portal vein, inferior vena cava, and hepatic vein was performed, and the 3D image was created. (**b**) Segmentation of each liver segment based on portal vein and its branches was performed. The fusion image with the iodine map of the left medial segment is shown in (**c**).

The liver volume was measured by a radiologist (four years of experience in abdominal radiology) on a workstation with a volume analyzer software program (SYNAPSE VINCENT version 7.0; Fujifilm Medical Corporation, Tokyo, Japan). The contours of the liver on blended 120 kV CT images were automatically outlined by a fully automated 3D volumetric CT software program to create 3D CT images of the whole liver. Large vessels, such as the portal vein, inferior vena cava, and hepatic vein, were then extracted semi-automatically in the PVP and EP, and the boundary of each hepatic segment was determined and color-coded using the extracted portal vein and hepatic vein as indicators (Figure 2). Based on the Couinaud classification of liver anatomy, the liver was divided into the caudate, left lateral, left medial, right anterior, and right posterior segments.

Next, the color-coded hepatic segments were extracted from the 3D CT images and separately superimposed on the iodine map images of the PVP and EP to calculate the iodine content of each hepatic segment (Figure 2). Misalignments in the superimposed images were manually corrected as necessary. Finally, the mean iodine concentration (mgI/cm^3^) of each hepatic segment was calculated by dividing the volumetric iodine content of each hepatic segment by the hepatic volume of each segment.

In addition, the ECV fraction of each hepatic segment was calculated using EP iodine map images. The calculation method was based on the following formula:

ECV fraction = (ICliver/ICaorta) × (1 − hematocrit)

where ICliver and ICaorta are the iodine concentrations in the hepatic segment and aorta, respectively, in the EP iodine map images.

The iodine concentration and ECV fractions were measured in the lateral, medial, anterior, and posterior segments of the liver. The caudate lobe was not evaluated in this study because it was sometimes difficult to accurately and automatically delineate its boundaries on superimposed iodine map images due to its complex structure consisting of the Spiegel lobe, paracaval portion, and caudate process [3].

After image processing and calculation, the volume, iodine concentration, and ECV fractions of each hepatic segment in the PVP and EP were compared between the cirrhotic and non-cirrhotic groups. The difference in iodine concentration between PVP and EP (ICPVP-liver—ICEP-liver) was also evaluated in each hepatic segment.

### 2.4. Statistical Analyses

All data were statistically analyzed by applying IBM SPSS software program (version 27.0; IBM, Armonk, NY, USA). Continuous variables were expressed as the mean ± standard deviation. In patient demographic characteristics, the Mann–Whitney U test and chi-square test were used between the cirrhosis and non-cirrhosis groups. The Mann–Whitney U test was used to compare the iodine concentration and ECV between the cirrhosis and non-cirrhosis groups. The iodine concentration and ECV of the liver parenchyma were compared among each hepatic segment using Friedman’s test followed by post hoc Dann test with Bonferroni correction. For predicting liver cirrhosis in each segment, the areas under the receiver operating characteristic (ROC) curve (AUC) were calculated. Cutoff values were obtained utilizing the maximized Youden index method based on the ROC analysis. Differences were considered statistically significant at *p* < 0.05.

## 3. Results

The results of the comparison of the patients’ demographic characteristics are shown in Table 1. No significant differences were found between the cirrhotic and non-cirrhotic groups with regard to age (*p* = 0.662) and sex (*p* = 0.806). There was also no significant difference in the ALBI score between the groups (*p* = 0.359) although the FIB-4 index (4.6 ± 4.2 vs. 1.7 ± 1.1) and APRI score (1.47 ± 1.94 vs. 0.52 ± 0.97) were significantly higher in the cirrhosis group than in the non-cirrhosis group (*p* < 0.001).

In the comparison of hepatic segmental volume, the cirrhosis group had a significantly greater volume of the lateral segment than the non-cirrhosis group ([259 ± 133] vs. [188 ± 49] ml, *p* < 0.05) and significantly smaller volume of the medial and anterior segments than the non-cirrhosis group ([114 ± 43] vs. [137 ± 34] ml, *p* < 0.05; [337 ± 128] vs. [405 ± 118] ml, *p* < 0.05) (Table 2).

**Figure 3:**
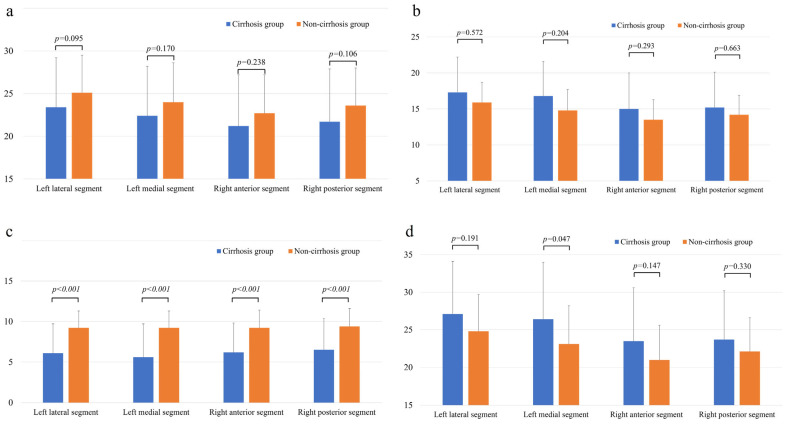
Comparison of iodine concentration (mgI/mL) in each hepatic segment in the PVP (**a**) and EP (**b**), the difference between PVP and EP in iodine concentration in each hepatic segment (**c**) and the ECV (%) per segment (**d**) between the cirrhosis group and the non-cirrhosis group. Note—PVP, portal venous phase; EP, equilibrium phase; and ECV, extracellular volume.

The comparison of iodine concentrations (mgI/cm^3^) in each hepatic segment is shown in Figure 3. In the PVP, the iodine concentration in the cirrhosis group tended to be lower than that in the non-cirrhosis group in all the hepatic segments. However, none of these differences were statistically significant (Figure 3a). In EP, the iodine concentration tended to be greater in the cirrhosis group than in the non-cirrhosis group in all segments, but the difference was not significant (Figure 3b). Conversely, the difference in iodine concentration (ICPVP-liver—ICEP-liver) between PVP and EP was significantly smaller in the cirrhosis group than in the non-cirrhosis group for all hepatic segments (*p* < 0.001) (Figure 3c). The AUC, cutoff value, sensitivity and specificity for predicting liver cirrhosis in each segment based on the difference in iodine concentration between the PVP and EP are shown in Table 3.

The results of the comparison of the ECV fractions in each hepatic segment are shown in Figure 3d. In the ECV fraction calculated for the EP iodine map images, the ECV fraction of the left medial segment was significantly higher in the cirrhosis group than in the non-cirrhotic group ([26.4 ± 7.6] vs. [23.1 ± 5.1]; *p* < 0.05).

Regarding the differences in iodine concentration and ECV between each hepatic segment, the iodine concentration in the lateral segment was significantly higher than that in the right anterior and posterior segments in both the cirrhosis and non-cirrhotic group (*p* < 0.001–0.016) (Table 4, Table 5 and Table 6). The ECV in the lateral segment was significantly higher than that in the medial and right anterior segments in both the cirrhotic and non-cirrhotic groups (*p* < 0.001) (Table 7).

## 4. Discussion

In the present study, each liver segment was successfully color-coded and extracted using a fully automated 3D volumetric CT program in contrast-enhanced dynamic images, which were then superimposed with iodine map images generated from DECT data to evaluate the iodine concentration and ECV fraction of each liver segment on a volumetric basis. This study showed that the cirrhosis group had a significantly greater volume in the lateral segment and significantly smaller volume in the medial and anterior segments than the non-cirrhosis group, findings consistent with previous reports of selective atrophy and compensatory enlargement, which are characteristic morphological changes in cirrhosis [9,10].

In contrast, the cirrhosis group exhibited a tendency towards lower iodine concentrations in the PVP across all segments, indicative of reduced portal venous flow. Conversely, the iodine concentrations in the EP and ECV fractions were greater, suggesting increased hepatic interstitial spaces, possibly due to fibrosis, compared with the non-cirrhosis group, but these differences were not significant, except for the ECV fraction in the medial segment. In this study, significant differences were observed between the two groups in the FIB-4 index and APRI, which serve as indicators of fibrosis, but not in the ALBI score, which is a marker of hepatic reserve capacity. These findings indicate that the degree of fibrosis may be underestimated in cases of cirrhosis with a preserved hepatic reserve capacity when the iodine concentration or ECV fraction is evaluated alone.

Conversely, the difference in iodine concentration between PVP and EP (ICPVP-liver—ICEP-liver) was significantly lower in the cirrhosis group than in the non-cirrhosis group, which is thought to be the result of the combined effect of reduced portal venous blood flow and increased interstitial space associated with fibrosis, allowing it to more sensitively reflect mild fibrosis and likely be a clinically useful indicator for assessing compensated cirrhosis. Further studies would be necessary to apply this method to early-stage fibrosis grading or longitudinal follow-up to evaluate its clinical utility.

In addition, when comparing the iodine concentration and ECV (%) in the PVP and EP between segments, the lateral segment was larger than the medial and anterior segments in both the cirrhotic and non-cirrhotic groups. One potential explanation for this phenomenon may be the predominance of splenic venous blood flow in the portal vein during periods of fasting, which tends to flow into the left lobe in cirrhosis [4,11]. The observation that analogous results were exhibited in the non-cirrhosis group is noteworthy, as it implies that, even in normal livers, the portal blood flow and the vascular bed are abundant in the left lobe.

Another reason for this result is the timing of the acquisition of EP images. The EP is generally defined as the time approximately 180 to 200 s after the intravascular and extracellular fluid contrast medium concentrations reach equilibrium [12], but there are various reports on the evaluation timing of liver fibrosis using equilibrium phase CT images, including evaluations at 3 min [8,13,14], 4 min [15,16], 10 min [17,18], and 30 min [19], so the optimal and precise time setting for evaluating liver fibrosis has not been clarified [20]. Compared with previous reports, the effects of blood flow may yet remain in the EP images taken after 3 min used in this study. In this case, it is presumed that the iodine concentration in the EP and ECV fractions was greater in the lateral segment than in the medial and anterior segments, reflecting the effect of blood flow.

Several limitations associated with the present study warrant mention. First, it may have been biased because of its retrospective design. Second, the overall sample size was small, particularly among patients with advanced cirrhosis and high ALBI and Child–Pugh grades. This was primarily due to the fact that many of the eligible patients during the study period had been scanned using other CT systems that lacked a dual-energy mode, which prevented their inclusion in the analysis. In addition, advanced cirrhosis itself is relatively uncommon in our clinical practice, which inherently limited the number of such cases. Future studies should include cirrhotic patients with larger sample sizes to enhance the statistical power and generalizability of the findings. Third, the diagnosis of cirrhosis was based on clinical manifestations without histological evidence, thus making it impossible to compare with the degree of histological fibrosis. Finally, the ECV fraction and iodine concentration in the EP were examined using CT images taken 3 min after contrast in this study. As noted above, further studies will be needed to determine the appropriate timing for assessing liver fibrosis.

## 5. Conclusions

The decreased difference in iodine concentration between PVP and EP calculated from 3D volumetric iodine mapping of the liver segment using DECT may be a clinically useful indicator for evaluating patients with compensated cirrhosis, suggesting a combined effect of a reduced portal venous flow and increased interstitial space associated with fibrosis.

## Figures and Tables

**Table 1:** Patient demographic characteristics.

Variable	All Patients (*n* = 66)	Cirrhosis Group (*n* = 34)	Non-Cirrhosis Group (*n* = 32)	*p* Value
Age (years)	65.2 ± 11.6	65.2 ± 12.9	65.3 ± 10.2	0.662
Sex *				0.800
Male	34	17	17	
Female	32	17	15	
AST	46.4 ± 78.5	57.3 ± 91.2	34.8 ± 61.6	<0.001
PLT	19.1 ± 7.9	14.8 ± 7.4	23.7 ± 5.7	<0.001
FIB-4 index	3.2 ± 3.4	4.6 ± 4.2	1.7 ± 1.1	<0.001
ALBI score	−2.6 ± 0.5	−2.5 ± 0.7	−2.8 ± 0.3	0.359
ALBI grade *		1 = 23, 2 = 7, 3 = 4	1 = 24, 2 = 8, 3 = 0	
Child-Pugh *		A = 27, B = 7, C = 0	N/A	
APRI	1.01 ± 1.61	1.47 ± 1.94	0.52 ± 0.97	<0.001

Unless otherwise specified, data are means ± standard deviations. * Data are numbers of participants. Note—AST, aspartate aminotransferase; PLT, platelet; FIB-4, fibrosis-4; ALBI, albumin–bilirubin; and APRI, aminotransferase/platelet ratio index.

**Table 2:** Comparison of hepatic segmental volume (mL) between the cirrhosis group and the non-cirrhosis group.

	Cirrhosis Group (*n* = 34)	Non-Cirrhosis Group (*n* = 32)	*p* Value
Left lateral segment	259 ± 133	188 ± 49	0.021
Left medial segment	114 ± 43	137 ± 34	0.010
Right anterior segment	337 ± 128	405 ± 118	0.019
Right posterior segment	316 ± 122	305 ± 80	0.964

Data are means ± standard deviations.

**Table 3:** AUC, cutoff value, sensitivity and specificity for predicting liver cirrhosis in each segment based on the difference in iodine concentration between the PVP and EP.

	AUC (95% CI)	Cutoff	Sensitivity	Specificity
Left lateral segment	0.759 (0.641–0.878)	5.95	0.50	1.00
Left medial segment	0.764 (0.644–0.883)	6.64	0.62	0.94
Right anterior segment	0.758 (0.639–0.878)	6.17	0.56	0.97
Right posterior segment	0.737 (0.611–0.863)	6.87	0.59	0.94

Note—AUC, area under the curve; CI, confidence interval.

**Table 4:** Comparison of iodine concentration (mgI/mL) in each hepatic segment in the PVP between the cirrhosis group and the non-cirrhosis group.

	Left Lateral Segment (LL)	Left Medial Segment (LM)	Right Anterior Segment (RA)	Right Posterior Segment (RP)	*p* Value						
					Overall	Pairwise					
						LL vs. LM	LL vs. RA	LL vs. RP	LM vs. RA	LM vs. RP	RA vs. RP
Cirrhosis group (*n* = 34)	23.4 ± 5.8	22.4 ± 5.8	21.2 ± 6.0	21.7 ± 6.2	<0.001	0.051	<0.001	<0.001	0.029	0.545	1.000
Non-cirrhosis group (*n* = 32)	25.1 ± 4.4	24.0 ± 4.6	22.7 ± 4.5	23.6 ± 4.4	<0.001	0.022	<0.001	0.006	0.001	1.000	0.006

Data are means ± standard deviations. Note—PVP, portal venous phase.

**Table 5:** Comparison of iodine concentration (mgI/mL) in each hepatic segment in the EP between the cirrhosis group and the non-cirrhosis group.

	Left Lateral Segment (LL)	Left Medial Segment (LM)	Right Anterior Segment (RA)	Right Posterior Segment (RP)	*p* Value						
					Overall	Pairwise					
						LL vs. LM	LL vs. RA	LL vs. RP	LM vs. RA	LM vs. RP	RA vs. RP
Cirrhosis group (*n* = 34)	17.3 ± 4.9	16.8 ± 4.8	15.0 ± 5.0	15.2 ± 4.9	<0.001	1.000	<0.001	<0.001	<0.001	0.001	0.362
Non-cirrhosis group (*n* = 32)	15.9 ± 2.8	14.8 ± 2.9	13.5 ± 2.8	14.2 ± 2.7	<0.001	0.006	<0.001	<0.001	<0.001	0.878	0.012

Data are means ± standard deviations. Note—EP, equilibrium phase.

**Table 6:** Comparison of the difference between PVP and EP in iodine concentration in each hepatic segment between the cirrhosis group and the non-cirrhosis group.

	Left Lateral Segment (LL)	Left Medial Segment (LM)	Right Anterior Segment (RA)	Right Posterior Segment (RP)	*p* Value						
					Overall	Pairwise					
						LL vs. LM	LL vs. RA	LL vs. RP	LM vs. RA	LM vs. RP	RA vs. RP
Cirrhosis group (*n* = 34)	6.1 ± 3.6	5.6 ± 4.1	6.2 ± 3.6	6.5 ± 3.9	<0.001	1.000	<0.001	0.016	<0.001	0.016	0.797
Non-cirrhosis group (*n* = 32)	9.2 ± 2.1	9.2 ± 2.1	9.2 ± 2.2	9.4 ± 2.2	<0.001	0.121	<0.001	0.001	0.001	0.728	0.121

Data are means ± standard deviations. Note—PVP = portal venous phase; EP, equilibrium phase.

**Table 7:** Comparison of the ECV (%) per segment between the cirrhosis group and the non-cirrhosis group.

	Left Lateral Segment (LL)	Left Medial Segment (LM)	Right Anterior Segment (RA)	Right Posterior Segment (RP)	*p* Value						
					Overall	Pairwise					
						LL vs. LM	LL vs. RA	LL vs. RP	LM vs. RA	LM vs. RP	RA vs. RP
Cirrhosis group (*n* = 34)	27.1 ± 7.0	26.4 ± 7.6	23.5 ± 7.1	23.7 ± 6.5	<0.001	<0.001	<0.001	<0.001	0.446	0.003	0.545
Non-cirrhosis group (*n* = 32)	24.8 ± 4.9	23.1 ± 5.1	21.0 ± 4.6	22.1 ± 4.5	<0.001	<0.001	<0.001	0.030	0.878	0.878	0.022

Data are means ± standard deviations. Note—ECV, extracellular volume.

## Data Availability

The datasets generated or analyzed during the study are available from the corresponding author upon reasonable request due to ethical restrictions; access requires approval from the institutional ethics committee.

## Abbreviations

The following abbreviations are used in this manuscript:

ALBI	albumin–bilirubin
AP	arterial phase
APRI	aspartate aminotransferase to platelet ratio index
CT	computed tomography
DECT	dual-energy computed tomography
ECV	extracellular volume
EP	equilibrium phase
FIB-4	fibrosis-4
PVP	portal venous phase
3D	three-dimensional

## References

[1] Gines P., Krag A., Abraldes J.G., Sola E., Fabrellas N., Kamath P.S. (2021). Liver cirrhosis. Lancet.

[2] Friedman S.L. (2015). Hepatic Fibrosis: Emerging Therapies. Dig. Dis..

[3] Ozaki K., Kozaka K., Kosaka Y., Kimura H., Gabata T. (2020). Morphometric changes and imaging findings of diffuse liver disease in relation to intrahepatic hemodynamics. Jpn. J. Radiol..

[4] Higaki A., Kanki A., Yamamoto A., Ueda Y., Moriya K., Sanai H., Sotozono H., Tamada T. (2023). Liver cirrhosis: Relationship between fibrosis-associated hepatic morphological changes and portal hemodynamics using four-dimensional flow magnetic resonance imaging. Jpn. J. Radiol..

[5] Jeon S.K., Joo I., Park J., Yoo J. (2024). Automated hepatic steatosis assessment on dual-energy CT-derived virtual non-contrast images through fully-automated 3D organ segmentation. Radiol. Med..

[6] Bak S., Kim J.E., Bae K., Cho J.M., Choi H.C., Park M.J., Choi H.Y., Shin H.S., Lee S.M., Kim H.O. (2020). Quantification of liver extracellular volume using dual-energy CT: Utility for prediction of liver-related events in cirrhosis. Eur. Radiol..

[7] McCollough C.H., Leng S., Yu L., Fletcher J.G. (2015). Dual- and Multi-Energy CT: Principles, Technical Approaches, and Clinical Applications. Radiology.

[8] Sofue K., Tsurusaki M., Mileto A., Hyodo T., Sasaki K., Nishii T., Chikugo T., Yada N., Kudo M., Sugimura K. (2018). Dual-energy computed tomography for non-invasive staging of liver fibrosis: Accuracy of iodine density measurements from contrast-enhanced data. Hepatol. Rrs..

[9] Ito K., Mitchell D.G. (2000). Hepatic morphologic changes in cirrhosis: MR imaging findings. Abdom. Imaging.

[10] Marti-Bonmati L., Delgado F. (2010). MR imaging in liver cirrhosis: Classical and new approaches. Insights Imaging.

[11] Tsukuda T., Ito K., Koike S., Sasaki K., Shimizu A., Fujita T., Miyazaki M., Kanazawa H., Jo C., Matsunaga N. (2005). Pre- and postprandial alterations of portal venous flow: Evaluation with single breath-hold three-dimensional half-Fourier fast spin-echo MR imaging and a selective inversion recovery tagging pulse. J. Magn. Reson. Imaging.

[12] Hwang J.A., Min J.H., Kang T.W., Jeong W.K., Kim Y.K., Ko S.E., Choi S.Y. (2021). Assessment of factors affecting washout appearance of hepatocellular carcinoma on CT. Eur. Radiol..

[13] Yoon J.H., Lee J.M., Kim J.H., Lee K.B., Kim H., Hong S.K., Yi N.J., Lee K.W., Suh K.S. (2021). Hepatic fibrosis grading with extracellular volume fraction from iodine mapping in spectral liver CT. Eur. J. Radiol..

[14] Nagayama Y., Kato Y., Inoue T., Nakaura T., Oda S., Kidoh M., Ikeda O., Hirai T. (2021). Liver fibrosis assessment with multiphasic dual-energy CT: Diagnostic performance of iodine uptake parameters. Eur. Radiol..

[15] Ito E., Sato K., Yamamoto R., Sakamoto K., Urakawa H., Yoshimitsu K. (2020). Usefulness of iodine-blood material density images in estimating degree of liver fibrosis by calculating extracellular volume fraction obtained from routine dual-energy liver CT protocol equilibrium phase data: Preliminary experience. Jpn. J. Radiol..

[16] Morita K., Nishie A., Ushijima Y., Takayama Y., Fujita N., Kubo Y., Ishimatsu K., Yoshizumi T., Maehara J., Ishigami K. (2021). Noninvasive assessment of liver fibrosis by dual-layer spectral detector CT. Eur. J. Radiol..

[17] Zissen M.H., Wang Z.J., Yee J., Aslam R., Monto A., Yeh B.M. (2013). Contrast-enhanced CT quantification of the hepatic fractional extracellular space: Correlation with diffuse liver disease severity. AJR Am. J. Roentgenol..

[18] Bottari A., Silipigni S., Carerj M.L., Cattafi A., Maimone S., Marino M.A., Mazziotti S., Pitrone A., Squadrito G., Ascenti G. (2020). Dual-source dual-energy CT in the evaluation of hepatic fractional extracellular space in cirrhosis. Radiol. Med..

[19] Bandula S., Punwani S., Rosenberg W.M., Jalan R., Hall A.R., Dhillon A., Moon J.C., Taylor S.A. (2015). Equilibrium contrast-enhanced CT imaging to evaluate hepatic fibrosis: Initial validation by comparison with histopathologic sampling. Radiology.

[20] Chandarana H., Shanbhogue K. (2021). Noninvasive Staging of Liver Fibrosis with Dual-Energy CT: Close but No Cigar. Radiology.

